# Enabling Factors for Sustaining Open Defecation-Free Communities in Rural Indonesia: A Cross-Sectional Study

**DOI:** 10.3390/ijerph14121572

**Published:** 2017-12-14

**Authors:** Mitsunori Odagiri, Zainal Muhammad, Aidan A. Cronin, Michael E. Gnilo, Aldy K. Mardikanto, Khaerul Umam, Yameha T. Asamou

**Affiliations:** 1 UNICEF Indonesia, World Trade Center 6 (10th Floor), Jalan Jenderal Sudirman Kav. 31, Jakarta 12920, Indonesia; acronin@unicef.org; 2 UNICEF Indonesia, Kupang Field Office, Gedung NTT Satu Data, Jl. Polisi Militer No. 2, Kupang 85111, Nusa Tenggara Timur, Indonesia; mzainal@unicef.org; 3 UNICEF, 3 UN Plaza, New York, NY 10017, USA; megnilo@unicef.org; 4 National Development Planning Agency (Bappenas), Government of Indonesia, Jl. Taman Suropati No. 2, Menteng, Jakarta 10310, Indonesia; amardikanto@gmail.com; 5 Planning, Research and Development Agency (Bapelitbang), Government of District of Alor, Jl. Bukit Doa Ayalon, Petleng, Alor Tengah Utara, Kabupaten Alor 85871, Nusa Tenggara Timur, Indonesia; umamkia3@gmail.com; 6 Alor District Health Office, Government of District of Alor, Jl. Profesor W.Z. Yohannes, Subo, Alor Selatan, Kabupaten Alor 85871, Nusa Tenggara Timur, Indonesia; yamehatgc@gmail.com

**Keywords:** Open Defecation Free (ODF) sustainability, latrine use, community approaches to total sanitation (CATS), social norms, Indonesia

## Abstract

Community Approaches to Total Sanitation (CATS) programmes, like the Sanitasi Total Berbasis Masyarakat (STBM) programme of the Government of Indonesia, have played a significant role in reducing open defecation though still little is known about the sustainability of the outcomes. We assessed the sustainability of verified Open Defecation Free (ODF) villages and explored the association between slippage occurrence and the strength of social norms through a government conducted cross-sectional data collection in rural Indonesia. The study surveyed 587 households and held focus group discussions (FGDs) in six ODF villages two years after the government’s ODF verification. Overall, the slippage rate (i.e., a combination of sub-optimal use of a latrine and open defecation at respondent level) was estimated to be 14.5% (95% CI 11.6–17.3). Results of multivariate logistic regression analyses indicated that (1) weaker social norms, as measured by respondents’ perceptions around latrine ownership coverage in their community, (2) a lack of all-year round water access, and (3) wealth levels (i.e., not being in the richest quintile), were found to be significantly associated with slippage occurrence. These findings, together with qualitative analysis, concluded that CATS programmes, including a combination of demand creation, removal of perceived constraints through community support mechanisms, and continued encouragement to pursue higher levels of services with post-ODF follow-up, could stabilize social norms and help to sustain longer-term latrine usage in study communities. Further investigation and at a larger scale, would be important to strengthen these findings.

## 1. Introduction

Globally, an estimated 892 million people still practice open defecation [1]. Poor sanitation leads to various infectious diseases such as diarrhea, soil-transmitted helminth, schistosomiasis, and trachoma infection [2,3,4]. Growing evidence suggests that poor sanitation is also associated with stunting and environmental enteropathy, resulting in increased risk of infectious disease, poorer cognitive development, lower educational outcomes at schools, and lower productivity in adult life [5]. Given the importance of sanitation for health and nutrition outcomes, the Sustainable Development Goals (SDGs) target 6.2 calls for ending open defecation and achieving universal access to sanitation, and also emphasizes equity, dignity, gender, and sustainability [1]. 

Amongst a number of established approaches for sanitation promotion, Community-Led Total Sanitation (CLTS) has expanded over recent years globally, as well as in East Asia [6,7,8]. CLTS is a non-subsidy approach that generates collective demand for sanitation within a community, and has proven effective for achieving Open Defecation Free (ODF) communities [6] by creating new social norms [9,10]. In 2008, UNICEF adopted the term Community Approaches to Total Sanitation (CATS), which is broader than CLTS in that it also encompasses the broader enabling environment that CLTS needs to achieve success, including legislation, financing, human resource capacity, supply chains, etc. with strong emphasis on sustainability and replication by involving Government from the start of the process [11]. The first cluster-randomised trial to evaluate a CLTS program in Mali has shown promising results with increased sanitation coverage and improved child growth [12]. There are, however, very few studies on the longer-term sustainability (i.e., sanitation outcomes several years after completion of programmes) of any type of sanitation interventions including CLTS programmes [13,14].

In Indonesia, it is estimated that the number of people still practicing open defecation ranges from 29 million [15] to 31 million [1], putting Indonesia in the top three highest burden countries for open defecation. High levels of stunting remain a challenge, and appear to be associated with poor sanitation and untreated drinking water [16]. With the national mid-term development plan, aiming for the elimination of open defecation by 2019, the Government of Indonesia has been making extensive efforts to achieve universal access to sanitation, including the acceleration of the Ministry of Health’s national sanitation programme, called STBM (Sanitasi Total Berbasis Masyarakat or Community-Based Total Sanitation in Indonesian language). The STBM programme follows the principles of CATS (i.e., demand creation for sanitation in communities to stop open defecation within a broader enabling environment), and consists of five pillars: to stop open defecation, promote handwashing with soap, improve household drinking water and food management, and manage solid and liquid waste [17]. These efforts have resulted in over 9000 verified ODF villages (i.e., achievement of the first pillar, to stop open defecation) in Indonesia [18]. However, like most of countries in this region, the current monitoring systems are not capable of capturing what happens in these ODF villages longer-term [8]. A better understanding of ODF sustainability and the dynamics of social norms is critical for informing sound post-ODF programming and for ensuring elimination of open defecation as per their national target.

Building on Alor district government efforts to evaluate their sanitation programme, this study used the government-collected data and examined the sustainability of ODF villages, as well as the intensity of empirical and normative expectations around latrine use behaviour as a proxy for social norms creation, in six ODF verified villages in Alor district, Nusa Tengara Timur (NTT) Province at least two years after a verification process by government. We further explored factors that are associated with slippage using both quantitative (i.e., a household survey) and qualitative data (i.e., Focus Group Discussions (FGDs)). Finally, implications of the study are discussed to inform the STBM programming efforts for improving ODF sustainability in Indonesia.

## 2. Materials and Methods

### 2.1. Study Area and Data Collection

**Figure 1:**
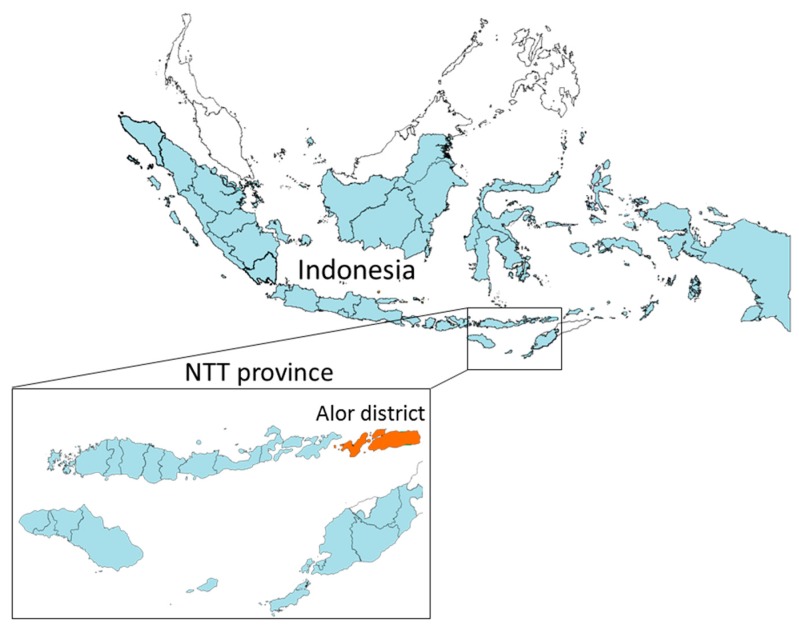
Overview map of Indonesia and the study area (Alor district, Nusa Tengara Timur (NTT) province).

Alor district, NTT province, is located in the eastern part of Indonesia (Figure 1), and consists of 175 villages with a population of 199,915 [19]. The poverty rate of Alor district is 20%, approximately twice the national figure (11%) [19]. Since 2013, the district government of Alor, with support from national and provincial governments and UNICEF, has been working to scale up STBM. The percentage of ODF verified villages, which achieves the first pillar of STBM (i.e., to stop open defecation), has increased substantially from 1% in 2014 to 61% in 2017 [18]. The proportions of households with access to improved sanitation (Indonesian Ministry of Health (MoH) definition of Jamban Sehat Permanen), basic sanitation (Indonesian MoH definition of Jamban Sehat Semi Permanen), and shared sanitation are 52.3%, 22.9% and 16.0%, respectively, in 2017 [18]. 

The Alor district government conducted a cross-sectional study and collected information on social expectations, access to sanitation, and latrine use behaviour from a total of 587 households that were randomly sampled in six villages, Village 1 (*n* = 77), Village 2 (*n* = 58), Village 3 (*n* = 77), Village 4 (*n* = 82), Village 5 (*n* = 153), and Village 6 (*n* = 140) in March 2017 as part of their sanitation programme evaluation. Villages were selected from 14 ODF verified villages in Pantar island, as these were among the first ODF verified villages in Alor district (details of village selection process in Supplementary Materials). We aimed to interview the head of households when available, resulting in 72% of respondents being the head of household. The sample size of households was calculated for a representative village-level population with 5% precision assuming a 15% slippage rate. Socio-demographic and WASH characteristics in study households are summarized in Table 1. All of the villages were triggered (i.e., STBM implementation) in 2013 and verified as ODF (i.e., achieving elimination of open defecation) by the District Health Office (DHO) in September to October 2014, following the MoH ODF verification guidelines [20] (details of criteria for verifying a community ODF in Supplementary Materials). 

To estimate the socioeconomic status of households, information on 11 assets was collected including radio, television, mobile phone, refrigerator, motorcycle, bicycle, gold jewelry, animal drawn cart, boat, agriculture land ownership, and farm animal ownership. Using these asset data, we performed a principal component analysis (PCA) for wealth score calculation, and classified households into wealth quintiles. All ethical and consent issues were part of the standard DHO data collection process in Alor district. The DHO standard data collection processes include pre-testing data collection tools, obtaining informed consent from all of the respondents and focus groups, and data anonymization before analysis. For ethical consideration, the Government body charged with community relations and public order (known as Badan Kesatuan Bangsa dan Politik in Indonesia) of the Government of Alor reviewed protocols and tools, and approved this programme evaluation. 

### 2.2. Household Slippage Measurement

**Figure 2:**
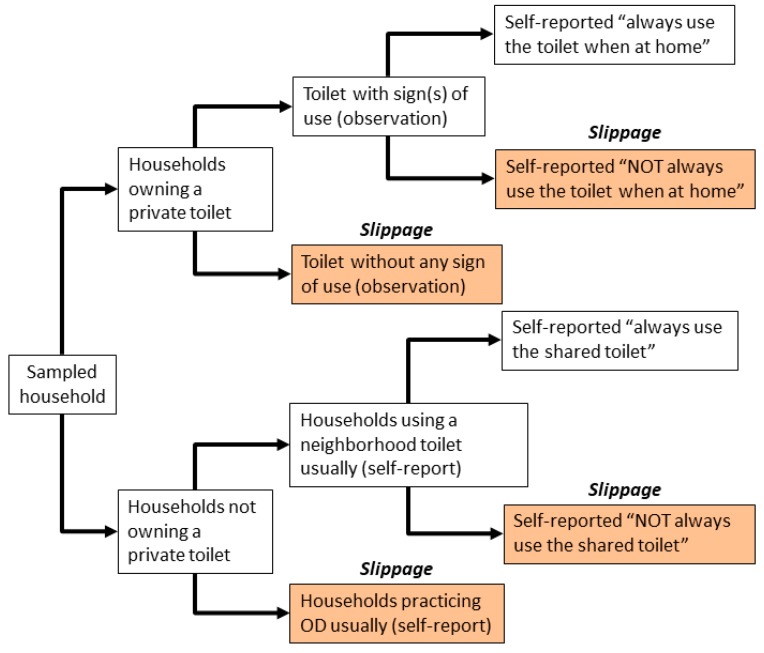
Flow diagram for identifying households that have slipped back based on observation and self-reported data of their latrine usage in this study.

Although the government ODF criteria cover a wider range of sanitation and hygiene practices, this study focused on consistent latrine use behaviour when household members were at home as a primary outcome of the STBM sustainability. We measured latrine usage via a combination of direct observation (i.e., latrine use at household-level) and self-reporting by respondents (i.e., latrine use at respondent-level) (Figure 2). For direct observation of household-level latrine use, six signs including (1) path to a latrine is walked on, (2) visibly used anal cleansing material observed, (3) if pour flush latrine, water is available, (4) detected feces in a pit using flash light, (5) slab is wet, and (6) smell in a toilet, were observed by enumerators. A latrine was considered being used by the household when at least one sign was observed at the time of survey. For self-reported latrine use, the respondents who own a private latrine or reported to use a neighbour’s latrine were asked how often they use their latrine when at home. Respondents chose one of the following answers, “Always”, “Usually/Mostly” “Sometimes/Occasionally”, and “Never” for latrine use. Slippage, or households classified as having slipped back to OD, included (1) those who reported to not have a private latrine and defecate in the open usually, (2) those who reported to not have a private latrine, and use a shared facility, but not always use the latrine, and (3) those who reported to have a private toilet, but not always use the toilet or households with a latrine not showing any sign of use via observation (Figure 2).

### 2.3. Social Norms Measurements

Based on Social Norms Theory, as defined by Bicchieri [9], we measured empirical and normative expectations regarding consistent larine use (See Supplementary Materials for details). Empirical expectations are about what a person expects other people will do, mostly based on that person experiences in the past of what he/she observed others did. Normative expectations are expectations about what a person thinks others expect him/herself to think or act. For measuring prevailing empirical and normative expectations, respondents were asked “Think about the people in your village, such as your family, friends, and neighbours. Out of 10 people in your village, how many do you think said that the members of their household always use a latrine?”, and “Think about the people in your village, such as your family, friends, and neighbours. Out of 10 people in your village, how many do you think/said that people should use a latrine because it is the right thing to do?”, respectively. These answers were treated as a scale variable for subsequent regression analysis, while the proportion of respondents who replied eight or more people was calculated for village-level descriptive statistics. 

Additionally, a series of statements that were related to expectations and normative beliefs around defecation practice were shown to respondents. Statements included (1) ‘Most people in this community do not have a toilet’ (empirical expectations), (2) ‘People in your village should use a toilet’ (normative belief), (3) ‘A lot of people think it is too expensive to have a toilet in their house’ (factual belief), (4) ‘In this community its acceptable to defecate in the open’ (factual belief), (5) ‘It’s embarrassing when people can see others defecating in the open’ (normative belief), (6) ‘Most people feel ashamed to not have a toilet in their house’ (factual belief), and (7) ‘It’s not a problem defecating on the beach, or in a river’ (factual belief). Respondents were offered a Likert scale choice of five options (“Strongly agree”, “Agree”, “Neither agree nor disagree”, “Disagree”, and “Strongly disagree”), based on their level of agreement with each statement. For statistical analysis, the five options were pooled into a binary variable (i.e., “Strongly agree/Agree” or “Not”), and each statement as one binary variable was tested for subsequent statistical analysis (i.e., multivariate logistic regression models tested for outcome variables of slippage occurrence and latrine ownership). As a proxy for the presence of social norms, we also asked whether any type of sanction exists when someone is seen defecating in the open in their community, and finally whether they knew their village was verified as ODF.

### 2.4. Statistical Analysis

To examine the impact of strength of social norms on (1) slippage (i.e., sub-optimal use of a private and neighbour’s latrine as well as reversion to OD usually) among all of the sampled households, and (2) slippage among households owning their private latrine, two separate multivariate analyses were performed. In multivariate model construction, all social norms-related variables, as described earlier, were included in addition to socio-economic factors (i.e., gender, age of respondents, education level, presence of a child under five years old, wealth quintile, size of household, and all year round water access for household needs) (based on the approach previously described [21]). 

For analysis on consistent latrine use outcomes among households owning their private latrine, independent variables were selected based on a conceptual framework proposed by Jenkins et al. [22] in addition to the same socio-economic factors and social norms related variables described above. Briefly, we included facility quality perceptions, satisfaction with facility, and motivation to use the facility. As previously described in Jenkins et al. [22], respondents’ perceptions on toilet facility quality and satisfaction with toilet as a place to defecate were assessed with five options (“Excellent”, “Good”, “Fair”, “Poor”, and “Very poor”) and four options (“Very satisfied”, “Satisfied”, “Dissatisfied”, and “Very dissatisfied”), respectively, which respondents were asked to choose. For motivation to use their toilet facility, respondents were asked what motivates the family to use the facility in an open-ended question. Enumerators recorded each response in one of 13 pre-determined reasons (“to prevent disease”, “to be clean and healthy living in their home”, “convenience”, “to have privacy in using the facility”, “to be modern”, “to be accepted well by others (i.e., pride/status)”, “to avoid sharing the facility with others”, “to avoid disturbing others in using a shared facility”, “to avoid embarrassment/humiliation”, “to follow what everybody is doing in the community”, “was told it was a right thing to use the facility”, “don’t know”, and “others”), accordingly. Each motivation factor was then treated as a binary variable (“Mentioned” or “Not mentioned”), and was used for constructing multivariate logistic regression models as described elsewhere [21]. Furthermore, in order to better understand challenges to building a private latrine, we examined factors that were associated with latrine ownership, where both social norms and socio-economic factors were included. 

In all of the analyses, we used logistic regression with generalized estimated equations (GEE) and robust standard errors, adjusting for village-level clustering, and estimated odds ratio (OR) to examine the associations between explanatory variables and binary outcomes of slippage and latrine ownership (Yes/No) as defined above. Univariate analysis was first performed, and variables with a *p*-value < 0.20 were included in subsequent multivariate models. Based on backward elimination, only variables with a *p*-value of <0.10 were retained in the final models, adjusted for socio-economic factors which were retained regardless of their *p*-values. In the final models, multicollinearity was examined based on the tolerance values, which were all above 0.74, indicating that multicollinearity was not considered as concern in this study. Results of three final models excluding the socio-economic factors with *p*-value > 0.1 are provided in supplemental tables for examining the impact of these non-significant socio-economic factors. The results showed the exclusion of these factors did not change the findings (i.e., statistically significant factors still reached the *p* < 0.05 after excluding them). 

For comparison of proportions between villages, we used a χ^2^ test. All of the analyses were conducted in SPSS ver. 22 (SPSS Inc., Chicago, IL, USA). In all of the analyses, *p* < 0.05 was considered significant.

### 2.5. Focus Group Discussions (FGDs)

FGDs were conducted in each of six ODF verified villages where a household survey was also conducted. The FGDs assessed the quality of STBM processes and follow-up activities, and intensity of social norms and social capital/cohesion, which are reported to be key factors for sustainable outcomes of CLTS [8]. A discussion guideline for FGDs was developed and pre-tested in a village of Alor district. Four male and four female participants (i.e., a total of eight participants) were invited per village, including the head of village, religious leaders, a member of village parliament, and the Family Welfare Movement (known as PKK in Indonesia), consisting of local women’s groups (the term “a local women’s group” is used in this study, referring to PKK). These key figures were purposefully chosen and recruited in all of the villages to best capture the STBM implementation process, subsequent follow-up, and social norms creation. The duration of each FGD was approximately one hour, which was recorded and transcribed in Indonesian language. These transcripts were reviewed by two to three facilitators who led the discussion. A bilingual staff translated transcripts into English. The FGD transcripts were analysed by thematic ordering using the Microsoft excel software as described elsewhere [23]. Briefly, for each FGD transcript, each statement was highlighted along the following pre-determined themes; “STBM pre-triggering conditions”, “STBM triggering”, “Establishment of a follow-up team and their sanitation message dissemination mechanisms”, “Community support mechanisms for building/improving a latrine.”, “Community challenges to achieve ODF”, “Motivation factors to mobilize community”, “Key influencers”, and “Social norms creation”. Each highlighted text was then transferred to a row in a table in an Excel sheet. For qualitatively capturing social capital/cohesion in villages, information on the presence of community support mechanisms, and the involvement of local civic organizations (e.g., religious groups and local women’s groups) were particularly focused on [24]. 

## 3. Results

### 3.1. Sanitation Access and Levels of Slippage

**Figure 3:**
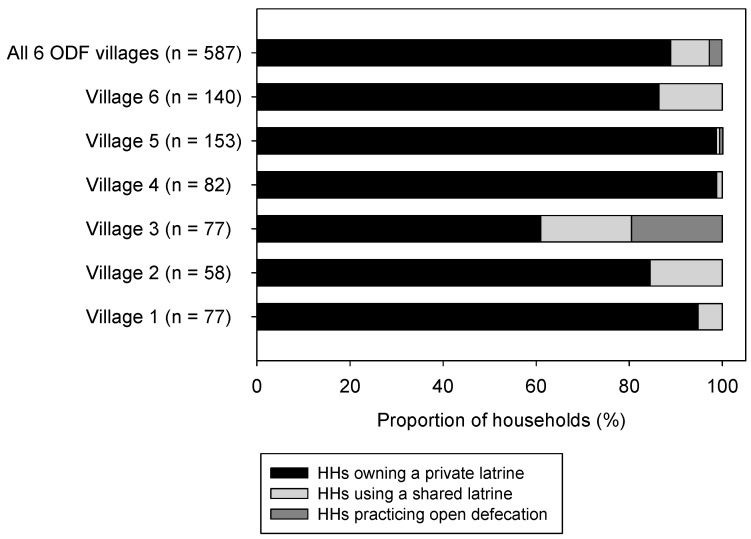
Proportion of households with access to a private latrine, and households without a private latrine but reporting to use a shared latrine, and reporting to practice open defecation most of times in six open defecation free (ODF) verified villages.

**Figure 4:**
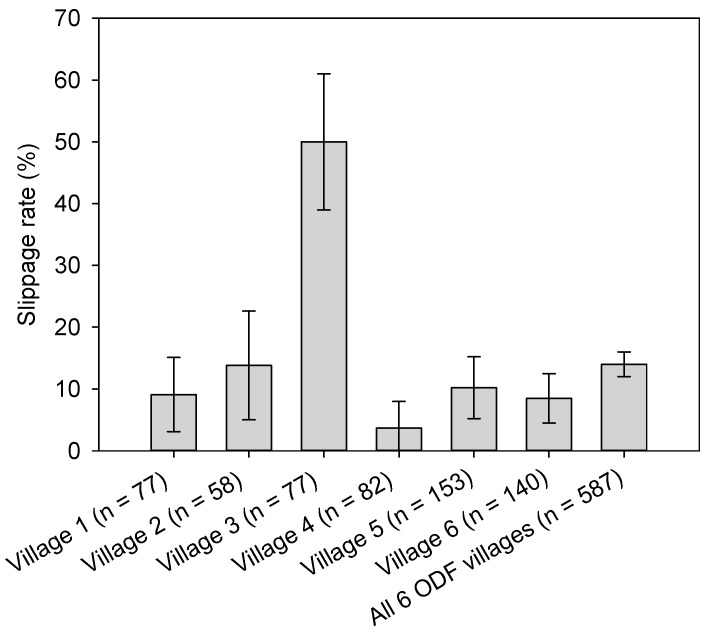
Slippage rates in six open defecation free (ODF) verified villages. The definition of slippage includes (1) households reporting to not always use their private latrine when at home, and households whose private latrine did not show any sign of latrine use via observation, (2) households reporting to not always use a shared latrine when at home, and (3) households reporting to practice open defecation usually. Error bars represent 95% confidence intervals.

In five of the six surveyed villages, over 85% (86.2–98.8%) of households had access to private sanitation facilities; whereas significantly fewer (61.2%, χ^2^ test, *p* < 0.05) of households in Village 3 had private latrine access. (Figure 3). Most private latrines were improved sanitation facilities (Table 1). The proportion of households not owning a private latrine and reporting to use a neighbour’s shared latrine usually ranged from 0.7 to 13.8% across all of the villages. Households that were reporting to practice open defecation usually were found in only two villages (Village 3: 19.5% (*n* = 15) and Village 5: 0.7% (*n* = 1)). Overall, the proportions of households owning a private latrine, households reporting to use a shared latrine, and households reporting to practice open defecation usually were 88.9% (95% CI 86.4–91.5, *n* = 522), 8.3% (95% CI 6.1–10.6, *n* = 49) and 2.7% (95% CI 1.4–4.0, *n* = 16), respectively. Levels of slippage were relatively similar in five ODF villages, ranging from 3.7 to 13.8%, with exception of Village 3 (51.9%) (Figure 4). On average, slippage was found to be 14.5% of households (95% CI 11.6–17.3, *n* = 85). Among respondents that were classified as having slipped back, 64.7% (95% CI 54.5–74.9, *n* = 55) owned a private latrine and 16.5% (95% CI 8.6–24.4, *n* = 14) used a shared toilet, not exclusively. The remaining 18.8% (95% CI 10.5–27.1, *n* = 16) reported to not own a private latrine, and to not use a shared toilet, but to practice open defecation usually (Figure S1 in Supplementary Materials). Slippage rate in the richest 20% of households was significantly lower when compared to the poorest and poorer, respectively (Table S1 in Supplementary Materials).

In the study villages, levels of normative and empirical expectations were found to be relatively high. Results of village-level social norms measurements are reported in Table 2. Overall proportions of respondents replying 8 or more out of 10 people for empirical and normative expectation proxies were 71.4% and 74.8%, respectively. Very small proportions of respondents agreed that most people in the community do not have a toilet (6.4%), it is acceptable to defecate in the open in the community (3.2%), and it is not a problem defecating on the beach or in a river (2.4%). Perceptions that there are existing sanctions for practicing open defecation in the community considerably varied between six ODF villages, ranging from 5.0 to 91.5%. Respondents’ recognition that their community was verified as ODF was widely observed in five ODF villages (i.e., 73.0–100%), but not in Village 3 (30.9%). In Village 3, where the highest slippage was observed among six study villages, perceptions around a private latrine ownership in their community, participation in a sanitation meeting, and recognition about their community as an ODF village, were significantly lower than those in the other five ODF villages (χ^2^ test, *p* < 0.05). Moreover, levels of empirical expectation were significantly lower than those of normative expectations as measured by proxy indicators in Village 3 (34.6% vs. 67.9%). Interestingly, a different trend was observed in Village 6 where the slippage rate was relatively low (7.9%), but both normative and empirical expectations were found to be low (33.1% and 39.0%, respectively).

### 3.2. Factors Associated with Overall Slippage (i.e., Households both Owning and Not Owning a Private Latrine) and Slippage among Households Owning a Private Latrine

In the multivariate logistic regression model exploring factors associated with overall slippage, respondents’ perceptions around latrine ownership in their community and acceptance of open defecation near a water body were found to be significant factors (Table 3). Specifically, respondents disagreeing that most people lack access to a toilet in their community and disagreeing that it is acceptable to defecate on the beach or in a river were significantly less likely to have slipped back (adjusted odds ratio (aOR) 0.36, 95% CI 0.19–0.67, and aOR = 0.44, 95% CI 0.21–0.92, respectively). Socio-economic factors that were significantly associated with slippage include wealth quintile and all year-round water access. 

For slippage in households owning a private latrine, there was statistical evidence that respondents that disagreed that most people do not have a toilet in their community are less likely to have slipped back (aOR 0.21, 95% CI 0.05–0.90) (Table 3). Motivation to use a latrine for cleaner and healthier living in their home was also found be associated with lower odds ratio of respondents having slipped back (aOR 0.50, 95% CI 0.30–0.81). Among the socio-economic factors that were tested, male respondents, a smaller size of households, being in the richest household quintile, and having access to all year-round water access for household needs were associated with lower odds of households having slipped back. There was also nearly significant evidence that satisfaction with a latrine as a place for defecation was associated with consistent latrine use behaviour (*p* = 0.066).

### 3.3. Factors Associated with Private Latrine Ownership

Looking at the association between social norms factors and private latrine ownership, we found evidence that respondents’ perceptions around latrine ownership, the perceived costs associated with latrine construction, acceptance of open defecation near to a water body, and latrine use behaviour in their communities were associated with latrine ownership (Table 4). Similar to two other models, the number of respondents disagreeing that most people do not have a toilet in their community was significantly associated with increased odds of households owning a private latrine (aOR 2.40, 95% CI 1.41–4.09). Respondents who disagree that a lot of people think it is too expensive to have a toilet in their house, and that it is acceptable to defecate on the beach or in a river were more likely to own a private latrine (aOR 3.25, 95% CI 1.16–9.1 and aOR 23.28, 95% CI 7.70–70.34, respectively). Empirical expectation levels (i.e., number of members of that household reporting to always use a latrine), were positively associated with increased odds of households owning a private latrine reflecting the importance of private sanitation on usage expectations. For socio-economic factors, the age of respondents (being above 51 years old), presence of a child under five years old, and wealth quintile (being in the richest) significantly increased OR for having a private latrine.

### 3.4. STBM Implementation and Process Assessment via Focus Group Discussions (FGDs)

Despite STBM being implemented by dedicated sanitation workers, based in a primary health center (Puskesmas in Indonesian language) in all of the villages, different levels of follow-up activities and community support mechanisms were identified between better-performing villages and the village with the highest slippage rate (i.e., Village 3). Key FGD findings are summarized in Table 5. (See Table S4 in Supplementary Materials for more details). 

In the better-performing villages, participants reported that messages around sanitation promotion and good hygiene had been consistently disseminated through mosques and churches in addition to their local STBM team in order to achieve ODF, while the engagement of religious platforms was not mentioned in Village 3. While religious leaders played a key role in the five villages, strong women’s involvement through the local women’s group, as well as the Posyandu (a monthly clinic for children and pregnant women) were also mentioned in raising awareness around the importance of a clean environment, latrine construction, and its usage in these same five villages. Looking at community-level support mechanisms as a proxy for social capital and cohesion, an informal revolving saving group (*arisan* in Indonesian language) and a mutual self-help mechanism (*gotong royong* in Indonesian language) where groups of people come and help to build a latrine were mentioned in the five villages, but not in Village 3. Furthermore, active post-ODF monitoring activities by members of local women’s groups and cadres were reported as part of efforts to achieve the additional pillars of STBM (i.e., to promote handwashing with soap, improve household drinking water and food management, and manage solid and liquid waste) in the five villages. These findings imply that STBM follow-up activities in both pre- and post-ODF status were more intense, and that social capital and cohesion were likely to be stronger in the better-performing villages, compared to Village 3.

In STBM implementation, commonly identified challenges around building a latrine included financial barriers to purchase non-locally-available materials for improved sanitation facilities, and insufficient water access for latrine use during dry seasons. However, these perceived financial constraints were reported to be overcome in some villages via community support mechanisms and some funding support from the village government. 

Finally, we observed suggestive evidence that most villages, excluding Village 3, created strong social norms around latrine use behavior. Participants mentioned “pride” that was associated with possessing an improved sanitation, “shame” attached to open defecation practices, and actions required for new households to build a latrine. One participant in Village 6 said that “although there is no defined sanction against open defecation practice in the community, the community has a strong commitment to stop open defecation, and people would lose their social standing if this commitment were violated”, which suggests the presence of informal social sanctions.

## 4. Discussion

### 4.1. ODF Sustainability, Slippage Pattern and Dynamics of Social Norms

We examined the sustainability of ODF village status (as measured by observed toilet usage and reported consistent latrine use behaviour) that was achieved through the national sanitation programme, STBM, in six ODF verified villages in rural Indonesia, using the government-collected data. We observed relatively low levels of respondent-level slippage in five ODF verified villages (8.8% on average), with exception of one village (Village 3: 51.9%). Similar levels of slippage rates have been reported one to two years after completion of CLTS programmes; for instance, from 17% of households practicing open defecation at the end of the intervention to 26% in one CLTS intervention in Ethiopia [13], from ODF status to 13% of households without a functional latrine in Ethiopia, Kenya, Uganda, and Sierra Leone [25], and from 20% of households without a sanitation facility at the end of the intervention to 31% in Mozambique [11]. These results, however, need to be compared with caution given the different definition of slippage, such as sub-optimal household-level latrine usage [13], households without a functional latrine [25], and sub-optimal individual-level latrine usage in this study. In addition, different follow-up periods and methodologies (i.e., comparison between a midline and enline condition or between ODF verification status and follow-up condition) were applied across the three studies. Standardized methodologies to measure latrine use behaviour will be useful for the comparability of the ODF sustainability outcomes of sanitation interventions in the future.

A plausible explanation for relatively low slippage rates observed in the five better-performing villages is that these villages are likely to have stronger community involvement with (1) high levels of social capital and cohesion, as measured by presence of community supporting mechanisms and existing civic organization engagements, and (2) active post-ODF follow-up. Previous CLTS studies in Indonesia and Ghana found that pre-existing levels of social capital may impact on the effectiveness and sustainability of CLTS [24,26,27]. In this study, FGDs revealed the presence of strong engagement of elected and religious leaders and local women’s group network together with community-level-self-support systems (i.e., *arisan* or *gotong royong* in Indonesian language) in achieving ODF status. These natural leaders were likely to have played a critical role to effectively influence the latrine adoption behaviour of community members within their reference network. Similarly, training natural leaders as part of a CLTS intervention has proven to be effective in Ghana [26]. This may be supported by findings in rural India where social network interactions significantly influenced decisions of latrine adoption [28]. 

This study reaffirms the importance of the presence of a post-ODF follow-up mechanism/protocol to gradually but systematically move communities to higher levels of service provision, e.g., towards achieving the complete five pillars of STBM in these villages. A study reviewing factors associated with sustained adoption of water, sanitation, and hygiene technologies reported that one of the most influential programme factors was frequent personal contact with a health promoter [29]. Developing a phased approach to higher levels of sanitation and hygiene service and building upon the social capital gained through ODF achievements are critical not only for sustaining sanitation outcomes, but for maximizing the health and nutrition benefits of comprehensive WASH and other health interventions [10,30]. 

Conversely, from FGDs in the village with the highest slippage rate, village members reported weak platforms for sanitation promotion, suggesting lower levels of social capital/cohesion in addition to financial barriers and severe water scarcity. Significantly less respondents reporting the attendance of a sanitation meeting would also suggest lower quality of STBM implementation. Despite ODF verification being an opportunity to reinforce social expectations as rewards [31], we observed in Village 3 that there were low levels of collective recognition that the community was ODF verified. Differences in the levels between observed normative and empirical expectations indicate that the majority of people in the community understood they should build and use a latrine, but failed to adopt and sustain the intended sanitation behaviours. Bicchieri and Xiao [32] reported that when normative and empirical expectations are in conflict, empirical expectations well predict decisions most likely due to dysfunctional punishment systems. This underscores that a combination of CLTS with support around strengthening the broader enabling environment, such as the CATS approach [11], is necessary for increasing the effectiveness and the sustainability of sanitation interventions. 

Interestingly, one village, Village 6, showed a low slippage rate (7.9%), but significantly lower levels of both normative and empirical expectations than the other four villages with low rates of slippage. Results of related statements around expectations and beliefs, such as recognition of the ODF status, and perceptions around community-level latrine ownership disagreed with these levels of expectations. However, FGDs revealed that social sanctions (i.e., losing their social stands) appeared to be strong, which may lead to lower slippage and may be important to track for post-ODF monitoring. Another plausible explanation is that given the relatively higher proportion of households using shared sanitation facilities that were observed in Village 6, there might be inconsistency between what respondents think people are doing in the community and what people are actually doing in the community. This might result in lower normative expectations despite the low slippage rate. Future research may strengthen these methodologies (i.e., questionnaire and reach) to better capture this issue.

### 4.2. Socio-Economic and Social Norms Related Factors Associated Overall Slippage among All Households, Slippage among Households Owning a Private Latrine, and Private Latrine Ownership

Three factors were consistently identified as being key drivers across the models: wealth level, all-year round water access for household needs, and respondents’ perceptions around latrine ownership in their community (i.e., most people in this community do not have a toilet, a proxy for empirical expectations). Economic barriers and water access during dry seasons were also confirmed as primary barriers in FGDs. Given lower private latrine ownership in the 40% poorest households (Table S1), it is likely that these households were reported to use a shared latrine during ODF verification. However, they failed to build a private latrine and were more likely to revert open defecation. Having seven or more household members was significantly associated with higher slippage occurrence among households owning a private latrine, which also suggests the challenges in sharing one latrine with several members. Appropriate support to the poorest is needed to help them move up the sanitation ladder. For challenges around water access, a pit latrine option might be more suitable in settings like the Alor district, where water is scarce during the dry season. However, strong preference to flush or pour-flush latrine and aspirations for higher levels of sanitation provision have been reported among households without latrines in rural Indonesia [21]. This suggests further efforts might be needed to introduce culturally-acceptable latrine options with lower water use requirements together with strengthening water access provision by local governments. Our findings around respondents’ perceptions on their community-level latrine ownership as a predictor of both consistent latrine use and private latrine ownership were in line with previous studies [13,14,21]. These findings suggest both normative and empirical expectations could be strengthened by reaching a tipping point of and/or gradual increasing sanitation coverage and use within their communities. Furthermore, recent discussions around minimum thresholds of community-level sanitation coverage (e.g., 60% or higher coverage) being necessary for achieving health impacts [33,34,35,36] could also be seen as social norms opportunities, as well as equity prerogatives, as underlined in the SDG target 6.2 [30]. Higher levels of sanitation access of surrounding households (i.e., reference network) are likely to strengthen both normative and empirical expectations, resulting in a higher likelihood of consistent latrine usage and meaningful health and nutrition gains. 

Looking at the gender aspects of latrine use behaviour among households with access to a private latrine, we found evidence that women respondents were more likely to report inconsistent use of the facility. It has been reported that women face gender-related cultural barriers to use a latrine in rural Zambia [37], and that women preferred not to use a latrine due to perceptions around cleanliness and smell inside a latrine in rural Ethiopia [38]. Conversely, other studies report opposite findings that women were more likely to use a latrine when they have access to a private latrine in rural India [39]. Further work is needed to better understand gender-related cultural dynamics around latrine use behaviour in rural Indonesia, but in this context, privacy, pride, and convenience were found to be important drivers for women in respect for building a latrine [21]. 

There was nearly significant evidence that lower levels of satisfaction with a latrine as a place to defecate was associated with inconsistent latrine use behaviour among respondents with access to a private latrine (*p* = 0.066). The majority of respondents who were dissatisfied with their latrine (*n* = 30, 88.2%) reported poor construction as the primary reason of facility dissatisfaction. This finding was consistent with previous studies [14,22,40], and additional support, such as enhancing financial flows from the national government and/or sanitation marketing to improve sanitation facilities, may be required to sustain their latrine use behaviour. Further investigation, on a larger scale, would be important to further test these findings and subsequently to support local STBM programs to design locally appropriate solutions.

### 4.3. Implications in Indonesia

Practical implications of this study findings in Indonesia are suggested as follows:

1. At national level, a systematic monitoring mechanism for assessing the sustainability of STBM outcomes, together with the development of associated guidance with clear indicators, will help to raise awareness to key decision makers, and for more efficient targeting and resource allocation. Measuring levels of social norms could help to better understand the sustainability of ODF status in communities.
2. There is a strong need for systematic approaches, including STBM, to achieve these objectives around ODF communities and improved sanitation coverage. Recent efforts in Indonesia to enhance fund flows for creating and improving sanitation access are welcome; examples are the use of zakat and wakaf (Islamic charity funds) [41], and also the Central Government instructions that the special allocation funds may be used for sanitation (known as DAK Sanitasi in Indonesia).
3. For the poorest, targeted smart financing schemes by village, sub-national or national governments may help to move them up the sanitation ladder from a shared latrine to a private latrine, and from a basic latrine to an improved. However, the demand creation process and collective action commitment to the elimination of open defecation by all of the community together should not be undermined, as per the principles of CATS/CLTS. Such systematic approaches may include regular monitoring and oversight from primary health centers (PUSKESMAS) and District Health Office (DHO), as well as all stakeholders working on a shared community action plan over a 3 to 5 year timeline, with key milestones agreed and owned by all.
4. Water access provision may be coupled with ODF achievements for sustaining latrine use behaviour. National and sub-national government may incentivize communities to achieve ODF as prerequisite for water provision.
5. This paper may inform monitoring and preventive measures to sustain ODF status. It is important for STBM to first focus on the achievement of ODF communities, and to then continue beyond ODF (i.e., to cover all five pillars of STBM). This requires continual support and demand generation efforts with all of the support of the entire community, especially to move open defecators onto the sanitation ladder, and to move those with basic or shared sanitation up to a higher level of WASH service provision and other community-based health interventions (e.g., reduction of stunting), and possibly in a phased fashion over a multi-year period. This will support communities to continuously improve their public health and child survival status by building on the new social capital and norms created via the achievement of an ODF community.

### 4.4. Limitations

This study has several important limitations. Firstly, latrine use behaviour was partly measured via self-reporting, which could exaggerate actual latrine use due to recall bias and social desirability. A survey question focusing on the 48 h prior to the date of the survey may possibly have been used to better captured latrine usage [42]. Similarly, responses on social norms around latrine use behaviour may be influenced by social desirability, and respondents might easily anticipate what enumerators expect to hear in ODF villages. These are common WASH research challenges, such as a combination of unblinded and use of a subjective outcome [43]. A method to incentivise correct answers may help in improving future accuracy. Secondly, slippage rates in this study may have been over-estimated due to our assumption that ODF status was truly achieved and confirmed during the ODF verification. This may not be always true if the ODF verification process was sub-optimal, as reported elsewhere [44]. Thirdly, social capital and cohesion in villages were qualitatively assessed in this study by examining the community support mechanisms and the involvement of local civic organizations through FGDs. This could have been improved applying a more robust methodology, such as construction of social capital indices [24] and a public standard good game [45]. Fourthly, our study was conducted in 6 ODF villages in Alor district, NTT province; our results may not be generalized to other parts of Indonesia. Fifthly, this cross-sectional study was conducted during the rainy season, and hence seasonal variability of latrine use behaviour could not be captured. Levels of sub-optimal latrine use might have been higher during dry seasons. Lastly, due to the nature of a cross-sectional study design, our study precludes identifying the direction of the causal effect between slippage occurrence and explanatory variables.

## 5. Conclusions

We investigated ODF sustainability and the associated dynamics around the creation and retention of social norms as part of the STBM programme evaluation in six ODF verified villages in rural eastern Indonesia two years after ODF verification. Our study found that latrine adoption and usage behaviour can be reasonably sustained for the longer-term, with (1) strong community engagement of natural leaders for reinforcing normative expectations, (2) community support mechanisms for removing constraints to acquire new behaviours and for reinforcing empirical expectations, and (3) continued encouragement to pursue higher level of services beyond ODF for stabilizing new social norms. 

Finally, as consistent with previous studies [13,14,24,46], this study confirmed that CATS might produce better sanitation outcomes, including sustainability, in settings where social capital and cohesion are stronger. Given the pressing need for achieving universal sanitation access as part of national and global SDGs targets, however, it points to the needs for more systematic and structured approaches to strengthen enabling environment that could accelerate ODF achievements and sustaining them at scale [30], including enhancing investment for processes that enhance community dialogue and the creation of social norms around open defecation. 

## Figures and Tables

**Table 1:** Household characteristics in six Open Defecation Free verified villages.

Characteristics	Proportions (%)					
	Village 1	Village 2	Village 3	Village 4	Village 5	Village 6
	(*n* = 77)	(*n* = 58)	(*n* = 77)	(*n* = 82)	(*n* = 153)	(*n* = 140)
Age of respondents						
18–37	48.1	20.3	30.9	32.9	32.0	29.6
38–50	26.0	27.1	38.3	50.0	32.7	27.5
>51	26.0	52.5	30.9	17.1	35.4	43.0
Sex of respondents						
Male	32.9	74.6	65.4	68.3	79.6	70.4
Female	67.1	25.4	34.6	31.7	20.4	29.6
Education level of respondents						
Not complete Primary	9.1	31.0	17.5	3.7	29.4	15.5
Primary	44.2	39.7	48.8	40.2	19.6	22.5
Pre-secondary	23.4	22.4	17.5	23.2	24.8	19.7
Secondary or higher	23.4	6.9	16.2	32.9	26.1	42.3
Size of households						
1–3	20.0	25.4	29.6	13.4	25.2	32.4
4–6	62.7	40.7	45.7	53.7	49.0	51.4
7 or more	17.3	33.9	24.7	32.9	25.8	16.2
Year-round water access for household use	98.7	94.8	42.9	100	73.2	100
Access to improved sanitation	96.1	86.2	55.8	98.8	92.8	86.4
Wealth quintile						
Richest	37.7	1.7	2.5	47.6	6.4	32.4
Richer	24.7	11.9	8.6	29.3	14	25.4
Middle	16.9	22	18.5	22.0	18.5	23.9
Poorer	19.5	37.3	30.9	0	14.6	16.9
Poorest	1.3	27.1	39.5	1.2	46.5	1.4

**Table 2:** Village-Level Social Norms around Latrine Use Behaviour in six Open Defecation Free (ODF) Verified Villages.

Questions/Statements	Type of Expectations/Beliefs	Proportions of Households in Percentage						
		Village 1 (*n* = 77)	Village 2 (*n* = 58)	Village 3 (*n* = 77)	Village 4 (*n* = 82)	Village 5 (*n* = 153)	Village 6 (*n* = 140)	All 6 ODF Villages (*n* = 587)
Think about the people in your village, such as your family, friends, and neighbours. Out of 10 people, how many do you think said that the members of their household always use a latrine? (More than or equal to 8)	Empirical expectations	90.7	76.3	34.6	100	93.4	39.0	71.4
Think about the people in your village, such as your family, friends, and neighbours. Out of 10 people, how many do you think said that people should use a latrine because it is the right thing to do? (More than or equal to 8)	Normative expectations	90.7	76.3	67.9	100	94	33.1 *	74.8
If someone in your village was observed defecating in the open, would any sanction happen to the person (Yes)	Not applicable	36	91.5	30.4	70.4	17.5	5	33.3
Most people in this community do not have a toilet. (Strongly agree/agree)	Empirical expectations	2.6	11.9	28.4 *	0	0.6	3.5	6.4
People in your village should use a toilet. (Strongly agree/agree)	Normative belief	97.4	93.2	92.6	98.8	96.1	93.7	95.3
A lot of people think it is too expensive to have a toilet in their house. (Strongly agree/agree)	Factual belief	13	18.6	32.5	1.2	3.9	37.3	18
In this community, it is acceptable to defecate in the open. (Strongly agree/agree)	Factual belief	0	5.1	2.5	1.2	6.5	2.1	3.2
It is embarrassing when people can see others defecating in the open. (Strongly agree/agree)	Normative belief	92.2	81.4	70.4	70.7	80	85.2	80.4
Most people feel ashamed to not have a toilet in their house. (Strongly agree/agree)	Factual belief	85.7	89.8	76.5	74.4	79.4	70.4	78
It is not a problem defecating on the beach, or in a river. (Strongly agree/agree)	Factual belief	5.2	0	4.9	1.2	0.7	2.8	2.4
Any household member participated in a meeting about sanitation or has any government staff visited your home to talk about sanitation. (Yes)	Not applicable	85.7	96.6	33.3*	69.5	71.6	63.1	68.4
I know that this community was verified as an ODF. (Yes)	Not applicable	93.5	100	30.9*	73	90.3	81	78.6

* indicates the proportion was significantly different from those of the other five villages (i.e., χ^2^ test, *p* < 0.05).

**Table 3:** Multivariate Logistic Regression Analysis on Factors Associated with (1) Slippage among All Households, and (2) Slippage among Households Owning a Private Latrine.

Factors	Slippage in all Households					Slippage in Households Owning a Private Latrine				
	N	Slippage Rate (%)	Adjusted OR	95% CI	*p*-Value	N	Slippage Rate (%)	Adjusted OR	95% CI	*p*-Value
Gender										
Male	402	13	0.61	(0.31–1.21)	0.159	357	8	0.50	(0.27–0.92)	0.025
Female	193	18	Ref			165	15	Ref		
Age (years)										
18–37	189	19	1.16	(0.77–1.75)	0.464	158	13	1.17	(0.50–2.75)	0.722
38–50	195	10	0.48	(0.36–0.63)	<0.001	172	7	0.47	(0.25–0.88)	0.018
>51	202	15	Ref			182	13	Ref		
Education										
Not complete Primary	109	16	1.11	(0.39–3.19)	0.846	93	12	0.91	(0.41–2.02)	0.820
Primary	191	18	1.12	(0.54–2.30)	0.762	159	12	1.18	(0.79–1.77)	0.417
Pre-secondary	128	13	0.94	(0.36–2.47)	0.907	116	10	1.12	(0.43–2.93)	0.819
Secondary or higher	162	11	Ref			149	9	Ref		
Size of households										
1–3	149	13	0.72	(0.39–1.32)	0.288	129	9	0.31	(0.15–0.64)	0.002
4–6	298	15	0.98	(0.79–1.23)	0.880	261	10	0.52	(0.29–0.91)	0.023
7 or more	141	15	Ref			124	13	Ref		
Presence of a child under 5 years old										
Yes	290	15	0.96	(0.69–1.33)	0.793	243	9	1.24	(0.83–1.85)	0.288
No	306	14	Ref			279	11	Ref		
Wealth quintile										
Poorest	124	23	2.36	(1.05–5.27)	0.037	101	14	3.85	(1.90–7.81)	<0.001
Poorer	109	19	2.26	(1.04–4.90)	0.039	84	13	3.33	(0.95–11.70)	0.061
Middle	122	11	1.37	(0.73–2.57)	0.324	109	9	2.08	(1.12–3.87)	0.020
Richer	115	16	2.68	(1.01–7.11)	0.048	108	14	4.46	(1.20–16.62)	0.026
Richest	127	5	Ref			120	4			
All year round water access for household needs										
Yes	498	11	0.60	(0.39–0.93)	0.021	450	10	0.49	(0.41–0.58)	<0.001
No	91	34	Ref			65	18	Ref		
Most people do not have a toilet.										
No	556	12	0.36	(0.19–0.67)	0.001	500	10	0.21	(0.05–0.90)	0.036
Strongly agree/agree	38	50	Ref			21	33	Ref		
It is not problem defecating on the beach or in a river.										
No	577	13	0.44	(0.21–0.92)	0.030					
Strongly agree/agree	14	23	Ref							
Satisfaction with a latrine										
Satisfied						461	10	0.32	(0.09–1.08)	0.066
Dissatisfied						34	26	Ref		
Cleaner and healthier living in our home										
Yes						389	8	0.50	(0.30–0.81)	0.006
No						133	17	Ref		
To avoid sharing with others										
Yes						35	29	15.41	(1.99–119.25)	0.009
No						487	9	Ref		

**Table 4:** Multivariate logistic regression analysis on factors associated with private latrine ownership.

Factors	Private latrine Ownership				
	N	Latrine Ownership (%)	Adjusted OR	95% CI	*p*-Value
Gender					
Female	191	89	Ref		
Male	403	86	0.70	(0.33–1.48)	0.349
Age (years)					
18–37	189	84	0.45	(0.24–0.84)	0.012
38–50	195	88	0.41	(0.22–0.76)	0.004
>51	201	91	Ref		
Education					
Not complete Primary	108	86	0.78	(0.22–2.78)	0.702
Primary	190	84	0.50	(0.26–0.99)	0.048
Pre–secondary	129	91	1.11	(0.84–1.49)	0.461
Secondary or higher	162	92	Ref		
Size of households					
1–3	149	87	0.42	(0.16–1.13)	0.087
4–6	297	88	0.95	(0.48–1.91)	0.895
7 or more	141	88	Ref		
Presence of a child under 5 years old					
Yes	290	84	3.31	(2.03–5.41)	<0.001
No	306	92	Ref		
Wealth quintile					
Poorest	125	81	0.15	(0.04–0.59)	0.007
Poorer	106	80	0.22	(0.05–0.88)	0.033
Middle	122	89	0.41	(0.25–0.66)	<0.001
Richer	115	94	0.86	(0.30–2.49)	0.785
Richest	127	94			
All year round water access for household needs					
Yes	498	91	1.76	(0.96–3.23)	0.067
No	90	72	Ref		
Most people do not have a toilet.					
Strongly agree/agree	38	55	2.40	(1.41–4.09)	0.001
No	556	90	Ref		
A lot of people think it is too expensive to have toilet in their house.					
Strongly agree/agree	107	68	Ref		
No	486	92	3.25	(1.16–9.13)	0.025
It is embarrassing when people can see others defecating in the open.					
Strongly agree/agree	477	90	Ref		
No	117	80	0.47	(0.20–1.08)	0.076
It is not problem defecating on the beach or in a river.					
Strongly agree/agree	14	36	Ref		
No	577	89	23.28	(7.70–70.34)	<0.001
How many do you think said that the members of their household always use a latrine (Scale: 0 to 10)					
			1.32	(1.19–1.46)	<0.001

**Table 5:** A summary of Focus Group Discussions (FGDs) in six Open Defecation Free villages.

**Theme**	**Village 1**	**Village 2**
STBM triggering	Facilitated by Puskesmas.	Facilitated by Puskesmas
Key message dissemination mechanisms after triggering to become ODF	The STBM team actively visited each household. Further sanitation promotion message was disseminated through church, mosque and community meetings.	The STBM team actively visited each household. Further message dissemination was done from church.
Presence of community support mechanisms to build and/or improve a latrine	Local support revolving fund (*Arisan*) & village government support to poorest household with some non-local materials.	Households were responsible to build a latrine with support from neighbors using “gotong royong” modality.
Key message dissemination mechanisms after ODF verification	Religious leaders and a local women’s group kept disseminating sanitation and other hygiene messages to motivate households.	Religious leaders kept disseminating messages. A local women’s group and cadres regularly visited households for hygiene promotion.
Community challenges to become ODF	Economic conditions of households affect latrine adoption. However, everyone support each other through *Arisan* and *Gotong royong*. The village government also provided financial/in-kind support.	Most people work as a farmer, and spend most their time in their fields, being unable to find time to build a latrine. Access to water is challenging during dry season.
Social norms creation	A local women’s group member said, “I believe that all people use a latrine. A few families still use a shared latrine, but I don’t see anyone defecating in the open. People in this village will feel ashamed to defecate in the open.”	All people in the community would say other think that all people should use a latrine to protect community health. People also feel comfortable using a latrine as it meets people’s privacy.
**Theme**	**Village 3**	**Village 4**
STBM triggering	Facilitated by Puskesmas	Facilitated by Puskesmas
Key message dissemination mechanisms after triggering to become ODF	Cadres continued to motivate the community. The head of village strongly encouraged community members to build a latrine.	Sanitation promotion messages were disseminated from mosques to improve latrines.
Presence of community support mechanisms to build and/or improve a latrine	No mechanism to support households to build or improve latrine was mentioned.	The poorest families received financial support from the District Government (~$40 USD) for improving a latrine.
Key message dissemination mechanisms to become ODF after triggering	No specific dissemination mechanisms were mentioned.	Religious leaders kept disseminating sanitation/hygiene promotion messages through mosques and churches
Community challenges to become ODF	The most challenging barrier is economic conditions of households who cannot afford to build an improved latrine. Water access during dry seasons is also a big barrier.	Most peoples in this village are farmers and working in the filed for a whole day. It is challenging to allocate their time to build a toilet.
Social norms creation	“A new family will build a latrine when building a house as all people in the community would feel ashamed if they did not have a latrine.”	“A new family will build a latrine when building a house as all people in the community would feel ashamed if they did not have a latrine.”
**Theme**	**Village 5**	**Village 6**
STBM triggering	Facilitated by Puskesmas	Facilitated by Puskesmas
Key message dissemination mechanisms after triggering to become ODF	Religious leaders promoted sanitation adoption and use through sermon. Community leaders and a local women’s group regularly visited households.	Sanitation promotion messages were disseminated from mosques. Cadres disseminated hygiene message to mothers.
Presence of community support mechanisms to build and/or improve a latrine	Households improved their toilet with *Arisan* and *Gotong royong* mechanisms.	*Gotong royong* was the main social capital for acceleration of sanitation promotion. For the poorest, financial support from the village was provided to buy cement for building a latrine
Key message dissemination mechanisms to become ODF after triggering	Community leaders and a local women’s group members kept disseminating sanitation/hygiene promotion messages.	Religious leaders kept disseminating sanitation promotion messages. Cadres also disseminated the hygiene practice messages to mothers.
Community challenges to become ODF	According to the village leader. “it is challenging for people to build an improve latrine because of lack of locally available materials. However, through *Arisan* and *Gotong royong*, people can overcome this.”	The poorest households are slow to build latrine. The village government provides in-kind support such as cements for latrine construction.
Social norms creation	A local women’s group member said, “We would feel ashamed and guilty if a guest saw a poor quality of a latrine. Having an improved latrine is a pride.”	All people in the community use a latrine because there is strong feeling of shame defecating in the open, pride of families owning an improved toilet as part of faith.

## Supplementary Materials

The following are available online at www.mdpi.com/1660-4601/14/12/1572/s1, Table S1: Slippage rates and private latrine ownership by wealth quintile, Table S2: Multivariate Logistic Regression Analysis excluding socio-economic factors with *p* value > 0.1 on Factors Associated with (1) Slippage among All Households, and (2) Slippage among Households Owning a Private Latrine, Table S3: Multivariate logistic regression analysis excluding socio-economic factors with *p* value > 0.1 on factors associated with private latrine ownership. Table S4: Summary of Focus Group Discussion (FGD) findings in 6 ODF verified villages, Figure S1: Relative proportion of household types among households that were classified as having slipped back.
