# Supplementary material for: Enabling Factors for Sustaining Open Defecation-Free Communities in Rural Indonesia: A Cross-Sectional Study
Source: Int J Environ Res Public Health. 2017 Dec 14;14(12):1572. doi: 10.3390/ijerph14121572 (PMC5750990; doi:10.3390/ijerph14121572)
Supplement: Supplementary file 1 [file ijerph-14-01572-s001.docx]

**Supplemental Material: Enabling Factors for Sustaining Open Defecation-Free Communities in Rural Indonesia: A Cross-Sectional Study**

Mitsunori Odagiri^1*^, Zainal Muhammad^2^, Aidan A. Cronin^1^, Michael E. Gnilo^3^, Aldy K. Mardikanto^4^, Khaerul Umam^5^ and Yameha T. Asamou^6^

**1. Village selection**

Six study villages were selected from 14 ODF verified villages on Pantar island as these were the earliest ODF verified villages in Alor district (please see details of village selection process in Supplemental Material). Fourteen ODF verified villages were further stratified by three categories; (1) costal, (2) mountainous and (3) in-between costal and mountainous areas to account for geographic challenges. However, only one village was located in costal and mountainous areas, respectively. Hence, the remaining 4 villages were randomly sampled from in-between costal and mountainous areas.

**2. Open Defecation Free (ODF) verification criteria defined by Ministry of Health (MoH)**

According to that verification guideline, the ODF verification criteria for pillar 1 (i.e. stopping open defecation) included (1) all household members defecate in a private or shared latrine, (2) a pit latrine has a lid or pour-flush latrine has a water seal for preventing flies coming in and out, (3) a latrine slab is made of strong and durable materials, (4) human faeces are not observed around houses and previous open defecation areas, (5) distance between final destination of human excreta (e.g. septic tank) and water sources is more than 10 m, (6) a latrine is emptied within 20 years of the installation, (7) infant’s and elder’s faeces are safely disposed of, and (8) a household has access to handwashing facility with water and soap.

**3. Social norms measurements**

Based on Social Norms Theory, expectations are about how respondents think about how others will think or act. Empirical expectations are about what a person expects other people will do, mostly based on that person experiences in the past of what he/she observed others did. They are not necessarily facts in the same way beliefs may not be facts. For example, a Japanese nod. A person from Japan might have an empirical expectation that when he meets someone new, that person will nod as a sign of respect. In reality, he might get a nod but he also might not. Normative expectations are expectations about what a person thinks others expect him/herself to think or act. Using the same example of the Japanese nod. A person from Japan might have a preference to nod when he meets someone new as a sign of respect because of his normative expectation.

**Table S1.** Slippage rates and private latrine ownership by wealth quintile. .

| Household type |  | N |  | Slippage in percentage (95%CI) |  | Private latrine ownership in percentage (95%CI) |
| --- | --- | --- | --- | --- | --- | --- |
| Poorest |  | 124 |  | 22.6 (15.2 - 29.9) |  | 80.8 (73.9 - 87.7) |
| Poorer |  | 109 |  | 19.3 (11.9 - 26.7) |  | 80.2 (72.6 - 87.8) |
| Middle |  | 122 |  | 10.7 (5.2 - 16.1) |  | 89.3 (83.9 - 94.8) |
| Richer |  | 115 |  | 15.7 (9.0 - 22.3) |  | 93.9 (89.5 - 98.3) |
| Richest |  | 127 |  | 4.7 (1.0 - 8.4) |  | 94.5 (90.5 - 98.5) |

**Table S2.** Multivariate Logistic Regression Analysis excluding socio-economic factors with P value > 0.1 on Factors Associated with (1) Slippage among All Households, and (2) Slippage among Households Owning a Private Latrine.

| **Factors** | **Slippage in all Households** | | | | |  | **Slippage in Households Owning a Private Latrine** | | | | |
| --- | --- | --- | --- | --- | --- | --- | --- | --- | --- | --- | --- |
|  | **N** | **Slippage rate (%)** | **Adjusted OR** | **95%CI** | **P-value** |  | **N** | **Slippage rate (%)** | **Adjusted**  **OR** | **95%CI** | **P-value** |
| Gender |  |  |  |  |  |  |  |  |  |  |  |
| Male |  |  |  |  |  |  | 357 | 8 | 0.47 | (0.25 – 0.89) | 0.020 |
| Female |  |  |  |  |  |  | 165 | 15 | Ref |  |  |
| Age |  |  |  |  |  |  |  |  |  |  |  |
| 18 – 37 | 189 | 19 | 1.26 | (0.87 – 1.84) | 0.229 |  | 158 | 13 | 1.10 | (0.48 – 2.49) | 0.823 |
| 38 – 50 | 195 | 10 | 0.53 | (0.40 – 0.71) | < 0.001 |  | 172 | 7 | 0.47 | (0.27 – 0.84) | 0.010 |
| > 51 | 202 | 15 | Ref |  |  |  | 182 | 13 | Ref |  |  |
| Education |  |  |  |  |  |  |  |  |  |  |  |
| Not complete Primary |  |  |  |  |  |  |  |  |  |  |  |
| Primary |  |  |  |  |  |  |  |  |  |  |  |
| Pre-secondary |  |  |  |  |  |  |  |  |  |  |  |
| Secondary or higher |  |  |  |  |  |  |  |  |  |  |  |
| Size of households |  |  |  |  |  |  |  |  |  |  |  |
| 1 – 3 |  |  |  |  |  |  | 129 | 9 | 0.33 | (0.13 – 0.85) | 0.022 |
| 4 – 6 |  |  |  |  |  |  | 261 | 10 | 0.55 | (0.30 – 1.00) | 0.050 |
| 7 or more |  |  |  |  |  |  | 124 | 13 | Ref |  |  |
| Presence of a child under 5 years old |  |  |  |  |  |  |  |  |  |  |  |
| Yes |  |  |  |  |  |  | 243 | 9 | 1.24 | (0.83 – 1.85) | 0.288 |
| No |  |  |  |  |  |  |  |  |  |  |  |
| Wealth quintile |  |  |  |  |  |  |  |  |  |  |  |
| Poorest | 124 | 23 | 2.61 | (1.05 – 6.51) | 0.040 |  | 101 | 14 | 3.96 | (1.77 – 8.87) | 0.001 |
| Poorer | 109 | 19 | 2.51 | (1.28 – 4.91) | 0.007 |  | 84 | 13 | 3.35 | (1.13 – 9.92) | 0.029 |
| Middle | 122 | 11 | 1.45 | (0.80 – 2.64) | 0.222 |  | 109 | 9 | 2.13 | (1.12 – 4.05) | 0.021 |
| Richer | 115 | 16 | 2.85 | (1.03 – 7.88) | 0.044 |  | 108 | 14 | 4.52 | (1.21 – 16.89) | 0.025 |
| Richest | 127 | 5 | Ref |  |  |  | 120 | 4 | Ref |  |  |
| All year round water access for household needs |  |  |  |  |  |  |  |  |  |  |  |
| Yes | 498 | 11 | 0.53 | (0.34 – 0.81) | 0.003 |  | 450 | 10 | 0.49 | (0.41 – 0.59) | < 0.001 |
| No | 91 | 34 | Ref |  |  |  | 65 | 18 | Ref |  |  |
| Most people do not have a toilet. |  |  |  |  |  |  |  |  |  |  |  |
| No | 556 | 12 | 0.30 | (0.15 – 0.57) | < 0.001 |  | 500 | 10 | 0.21 | (0.05 – 0.92) | 0.038 |
| Strongly agree/ agree | 38 | 50 | Ref |  |  |  | 21 | 33 | Ref |  |  |
| It is not problem defecating on the beach or in a river. |  |  |  |  |  |  |  |  |  |  |  |
| No | 577 | 13 | 0.45 | (0.24 – 0.83) | 0.011 |  |  |  |  |  |  |
| Strongly agree/ agree | 14 | 23 | Ref |  |  |  |  |  |  |  |  |
| Satisfaction with a latrine |  |  |  |  |  |  |  |  |  |  |  |
| Satisfied |  |  |  |  |  |  | 461 | 10 | 0.31 | (0.09 – 1.06) | 0.062 |
| Dissatisfied |  |  |  |  |  |  | 34 | 26 | Ref |  |  |
| Cleaner and healthier living in our home |  |  |  |  |  |  |  |  |  |  |  |
| Yes |  |  |  |  |  |  | 389 | 8 | 0.50 | (0.30 – 0.82) | 0.006 |
| No |  |  |  |  |  |  | 133 | 17 | Ref |  |  |
| To avoid sharing with others |  |  |  |  |  |  |  |  |  |  |  |
| Yes |  |  |  |  |  |  | 35 | 29 | 15.38 | (2.08 – 113.86) | 0.007 |
| No |  |  |  |  |  |  | 487 | 9 | Ref |  |  |

**Table S3.** Multivariate logistic regression analysis excluding socio-economic factors with P value > 0.1 on factors associated with private latrine ownership.

| Factors | Private latrine ownership | | | | |
| --- | --- | --- | --- | --- | --- |
|  | N | Latrine ownership (%) | Adjusted OR | 95%CI | P–value |
| Gender |  |  |  |  |  |
| Female |  |  |  |  |  |
| Male |  |  |  |  |  |
| Age |  |  |  |  |  |
| 18 – 37 | 189 | 84 | 0.47 | (0.28 – 0.77) | 0.003 |
| 38 – 50 | 195 | 88 | 0.48 | (0.25 – 0.92) | 0.026 |
| > 51 | 201 | 91 | Ref |  |  |
| Education |  |  |  |  |  |
| Not complete Primary | 108 | 86 | 0.94 | (0.24 – 3.64) | 0.926 |
| Primary | 190 | 84 | 0.59 | (0.29 – 1.22) | 0.155 |
| Pre–secondary | 129 | 91 | 1.26 | (0.95 – 1.68) | 0.108 |
| Secondary or higher | 162 | 92 | Ref |  |  |
| Size of households |  |  |  |  |  |
| 1 – 3 |  |  |  |  |  |
| 4 – 6 |  |  |  |  |  |
| 7 or more |  |  |  |  |  |
| Presence of a child under 5 years old |  |  |  |  |  |
| Yes | 290 | 84 | 2.62 | (1.29 – 5.30) | 0.008 |
| No | 306 | 92 | Ref |  |  |
| Wealth quintile |  |  |  |  |  |
| Poorest | 125 | 81 | 0.15 | (0.04 – 0.60) | 0.007 |
| Poorer | 106 | 80 | 0.22 | (0.06 – 0.89) | 0.033 |
| Middle | 122 | 89 | 0.43 | (0.29 – 0.63) | < 0.001 |
| Richer | 115 | 94 | 0.88 | (0.29 – 2.71) | 0.828 |
| Richest | 127 | 94 |  |  |  |
| All year round water access for household needs |  |  |  |  |  |
| Yes | 498 | 91 | 1.57 | (0.92 – 2.67) | 0.101 |
| No | 90 | 72 | Ref |  |  |
| Most people do not have a toilet. |  |  |  |  |  |
| Strongly agree/ agree | 38 | 55 | 2.06 | (1.34 – 3.17) | 0.001 |
| No | 556 | 90 | Ref |  |  |
| A lot of people think it is too expensive to have toilet in their house. |  |  |  |  |  |
| Strongly agree/ agree | 107 | 68 | Ref |  |  |
| No | 486 | 92 | 3.03 | (1.25 – 7.35) | 0.014 |
| It is embarrassing when people can see others defecating in the open. |  |  |  |  |  |
| Strongly agree/ agree | 477 | 90 | Ref |  |  |
| No | 117 | 80 | 0.48 | (0.21 – 1.07) | 0.074 |
| It is not problem defecating on the beach or in a river. |  |  |  |  |  |
| Strongly agree/ agree | 14 | 36 | Ref |  |  |
| No | 577 | 89 | 19.12 | (7.29 – 50.15) | < 0.001 |
| How many do you think said that the members of their household always use a latrine (Scale: 0 to 10) |  |  |  |  |  |
|  |  |  | 1.31 | (1.17 – 1.47) | < 0.001 |

**Table S4.** Summary of Focus Group **Discussion** (FGD) findings in 6 ODF verified villages.

| Theme | Village 1 | Village 2 | | Village 3 | |  |
| --- | --- | --- | --- | --- | --- | --- |
| Before STBM triggering | Prior to STBM, there was a programme called "village tourism" aiming to improve environment cleanliness. But, a small portion of people still practiced open defecation. | Most people defecated in forest, and only a few households had a latrine. | | Most people defecated in the open. | |  |
| STBM triggering | Facilitated by Puskesmas (Local Health centre) & District Health Office (DHO), The village leader improved the village programme with STBM. | Facilitated by Puskesmas | | Facilitated by Puskesmas | |  |
| STBM triggering participants | Most community members and leaders | Most community members attended the triggering | | Community leaders, school teachers, religious leaders and community | |  |
| STBM team established | Yes, the head of village did so. The team including a head of village, staff, religious leader, Women’s group (PKK), head of sub-village units (dusun, RW, RT) and local health promotion team (cadres) | Yes, the head of village did establish the team which included a village government staff, church, and cadres. | | A team consists of village government staffs and cadres working to follow up the triggering. | |  |
| Key message dissemination mechanisms to become ODF | The STBM team actively visited each Household (HH). | The STBM team actively visited each HH. | | Cadres continued to motivate the community | |  |
|  | Further sanitation message was disseminated through church, mosque and community meetings. | Further message dissemination was done from church | | Head of village strongly people build toilet. | |  |
| Any type of support mechanisms to build and/or improve a latrine | Local support revolving fund (Arisan) & village government support to poorest household with some non-local materials. | HHs were responsible to build a latrine with support from neighbors using "gotong royong" modality. | | No mechanism to support HHs to build or improve latrine | |  |
| Presence of Sanction | "Sanction is not needed as people in the community consistently use their latrine." | As of now, setting a sanction for preventing open defecation is not agreed, but the village is considering it. | | There is no sanction. | |  |
| ODF monitoring & verification | Monitoring & evaluation done by sanitarians and cadres. Verification was done twice in 2013 and 2014. District staff also conducted verification before declaration. | Puskesmas conducted monitoring and verification. District staff also verified the community before declaration. | | Puskesmas and sub-district WASH facilitator conducted the monitoring and verification. | |  |
| Key message dissemination mechanisms after ODF verification | Religious leaders keep disseminating messages and PKK also motivated HHs to adopt other hygiene practices. | Religious leaders keep disseminating messages. PKK and cadres conduct home visit to motivate the HHs. | |  | |  |
| Financial support mechanisms after ODF verification | Households improve a pit latrine to pour-flush latrine using arisan or village fund. |  | | No village fund allocation yet | |  |
| Challenges to become ODF | Economic conditions of households affected how soon they could build a latrine. But, everyone supported each other through arisan and gotong royong. Village government also provided support. | Most people work as farmers, and spend most their time in their fields, being unable to find sufficient time to build a latrine. Not-locally-available materials for improving a latrine is also a barrier. Access to water is challenging particularly during dry season. Old people, widow and new families had difficulty in building a latrine. | | The most challenging barrier is their economic conditions of households who cannot afford to build an improved latrine. Water access during dry seasons is also a big barrier. | |  |
| Motivation / EE to become ODF | Key motivation factors include the commitment of a head of village and support from all community leaders and cadres. The village leader said "all of us working together to make all people aware the importance of using a latrine and motivated them to build and use their own latrine". | A key motivation factor was a strong commitment of a village leader with support from religious leaders and cadres. | | A key motivation factor was leadership of a head of village. All people followed hi. | |  |
| Key influencers | Sub-district head (Camat), head of village, sanitarian and district staff | Village government, religious leaders and cadres | | Head of village | |  |
| Social norms creation - general | According to a PKK member, "I believe that all people use a latrine. A few families still use a shared latrine, but I don't see anyone defecating in the open. All people in this village will feel ashamed to defecate in the open." | All people in the community would say other think that all people should use a latrine to protect community health. People also feel comfortable using a latrine as it meets people's privacy. | | Most people have their own latrine and some use a neighborhood’s latrine. But, there are some households practicing open defecation. | |  |
| Social norms creation - defecation practice while working | Most families work as fishermen, and go back to their home for defecation as the workplace is not far from their home. | All people use a latrine when at home. However, when they are working in field, they defecate in forest near the field as there is a no public toilet in field. | | Most people are farmers, and defecate in the field when working there. | |  |
| Social norms creation - New families | New families, if any, should use the toilet, No space for OD because OD is not accepted anymore. | Newly migrated households will be encouraged to build a latrine by informing that all households use a latrine in the village. | | New families usually use their parent's latrine, but sometimes defecate in the open. | |  |
| Social norms creation - Children | School children also use a latrine in schools. | School children also use a latrine in schools. | |  | |  |
| Theme | Village 4 | | Village 5 | | Village 6 | |
| Before triggering - 2013 | Most HHs had a pit latrine, but not pour-flush latrines | | Despite earlier sanitation message dissemination, still several households defecated in the open (forest) | | Most people defecated in the open (field and beach) | |
| STBM triggering | Facilitated by Puskesmas with support from cadres | | Facilitated by Puskesmas | | Facilitated by Puskesmas | |
| STBM triggering participants | Community members was mobilized to attend the triggering. | | Most people in the village participated and attended by community leaders such as PKK, cadres, teachers, etc. | | Most people participated in the triggering because of a strong leadership of the village leader. PKK, cadres, community leaders and religious leaders actively supported the triggering session. | |
| STBM team established | Village government and cadres were working as a team to motivate HHs to build and use latrine | | Village government and cadres were working as a team to motivate HHs to build and use latrine | | Village government, PKK, cadres, RT and RW were working as a team to follow up the triggering. | |
| Key message dissemination mechanisms to become ODF | Message from mosques was disseminated to improve latrines | | Religious leaders announce through sermon to stop OD and improve the quality of latrines | | Message dissemination from mosques | |
|  |  | | Community leaders and PKK teams visited HHs regularly | | Cadres disseminated message to mothers (child health program), Jumat Bersih - Friday cleanliness drive) | |
| Any type of support mechanisms to build and/or improve a latrine | The poorest families received Housing Improvement allowance from District Government and IDR 500,000 (~$40 USD) of the amount was aimed to improve a latrine. | | Village fund are not allocated for improving a latrine. Head of village think that HHs can improve the toilet with Arisan & building on local social capital (Gotong royong) only. Having an improved toilet was became a pride. | | Gotong royong is the main social capital used to accelerate the ODF. For the poorest, stimulant was provided to build a latrine, such as to buy cement | |
| Presence of Sanction | "No sanction is set as all people feel ashamed practicing open defecation." | | There is a sanction agreed in the village. He/she needs to clean his/her feces if they practice open defecation, but we don't find anyone defecating in the open. | | There is no written sanction, but the community has strong agreement to stop open defecation. People will lose their social standing if they violate this agreement (i.e. to stop open defecation) | |
| ODF monitoring & verification | Puskesmas and sub-district WASH facilitator conducted the monitoring and verification. | | Verification was conducted by Puskesmas. A District team also conducted verification before declaration. | | Verification was conducted by Puskesmas and District health Office with village government, RT/RW and PKK. | |
| Key message dissemination mechanisms after ODF verification | Religious leaders keep disseminating messages through mosques and churches | | Community leaders and PKK cadres keep disseminating messages. | | Religious leaders keep disseminating messages that cleanses is a part of faith. Cadres also disseminated the hygiene practice messages to mothers. | |
| Financial support mechanisms after ODF verification |  | |  | |  | |
| Challenges to become ODF | Most peoples in this village are farmers and working in the filed for whole day. Difficult to allocate time to build toilet. | | According to the village leader. "it is challenging for people to build an improve latrine because it requires materials that cannot be obtained locally. However, through arisan and Gotong Royong, people can overcome this." | | The poorest HHs were slow to build latrine and for the reason, village government provided stimulant such as cements to them. | |
| Motivation / EE to become ODF | A key motivation factor was provision of housing allowance including IDR 500,000 for improving a latrine. | | A key motivation factor was people felt ashamed to have a pit latrine and wanted to improve it. | | A village programme called "one house, one toilet, latrine competition" motivated households to build a latrine. Village government has target each house has an improved toilet. | |
| Key influencers | Head of village, religious leaders and cadres | | Head of village and local village parliament), religious leaders and cadres | | Village government, Puskesmas, RT/RW, PKK and Dasa Wisma (a woman chair in the smallest unit of community (usually consist of 10 households) for PKK activities) | |
| Social norms creation - general | "A new family will build a latrine when building a house as all people in the community would feel ashamed if they did not have a latrine." | | According to the PKK member, "We would feel ashamed and guilty if a guest visited us and saw a poor quality of a latrine. Having an improved latrine is a source of pride." All people use a latrine and no one engages in open defecation any more. Some households own two latrines to serve all household members. | | All people in the community use a latrine because there is strong feeling of shame defecating in the open, pride of the family owning an improved toilet and cleanliness as part of faith. | |
| Social norms creation - defecation practice while working | Most people are seaweed farmers. They always use a latrine to keep their beach clean. A small portion of people are farmers, and defecate in the field when working there due to lack of a public latrine. However, they dig a hole and cover it after their defecation. They are aware that leaving human feces in the open will negatively affect community health. | | Most people are working as fishermen and have no space to do OD in the beach anymore. | | Most people are working as fishermen and have no space to do OD in the beach anymore. | |
| Social norms creation - New families |  | | When a new family has not built a latrine, they use a neighbors or their parent’s toilet. The village government requires new families to build a latrine as soon as possible. | | New families always use a public or neighborhood’s latrine until their latrine is built. | |
| Social norms creation - Children | School children also use a latrine in schools. | |  | |  | |

**Figure S1.** Relative proportion of household types among households that were classified as having slipped back. The definition of slippage includes (1) households reporting to not always use their private latrine when at home, and households whose private latrine did not show any sign of latrine use via observation, (2) households reporting to not always use a shared latrine when at home, and (3) households reporting to practice open defecation most of times.
